# Reading between Eye Saccades

**DOI:** 10.1371/journal.pone.0006448

**Published:** 2009-07-30

**Authors:** Caroline Blais, Daniel Fiset, Martin Arguin, Pierre Jolicoeur, Daniel Bub, Frédéric Gosselin

**Affiliations:** 1 Département de Psychologie, Université de Montréal, Montréal, Québec, Canada; 2 Department of Psychology, University of Victoria, Saanich, British Columbia, Canada; University of Sydney, Australia

## Abstract

**Background:**

Skilled adult readers, in contrast to beginners, show no or little increase in reading latencies as a function of the number of letters in words up to seven letters. The information extraction strategy underlying such efficiency in word identification is still largely unknown, and methods that allow tracking of the letter information extraction through time between eye saccades are needed to fully address this question.

**Methodology/Principal Findings:**

The present study examined the use of letter information during reading, by means of the Bubbles technique. Ten participants each read 5,000 five-letter French words sampled in space-time within a 200 ms window. On the temporal dimension, our results show that two moments are especially important during the information extraction process. On the spatial dimension, we found a bias for the upper half of words. We also show for the first time that letter positions four, one, and three are particularly important for the identification of five-letter words.

**Conclusions/Significance:**

Our findings are consistent with either a partially parallel reading strategy or an optimal serial reading strategy. We show using computer simulations that this serial reading strategy predicts an absence of a word-length effect for words from four- to seven letters in length. We believe that the Bubbles technique will play an important role in further examining the nature of reading between eye saccades.

## Introduction

Gutenberg's invention has democratized the written word to such an extent that it is estimated that nowadays the average English reader has been exposed to more than 100 million printed words before he reaches the age of 25 [1]. As a result of this intense exposure, readers become experts, and word identification is made rapidly and effortlessly, typically with no or little cost of the number of letters for words containing less than seven letters [2]–[4].

One fundamental issue on which current knowledge is lacking is the information extraction strategy underlying expert word identification, which is the focus of the present paper. Most of what we do know about the time course of reading comes from methods that infer the order of letter extraction from reaction times or overall accuracy. However, the outcomes of these studies has led to different hypotheses, ranging from spatially parallel to pure sequential letter processing. A method that allows the tracking of the useful visual information extraction more directly would help tease these hypotheses apart.

Eye-tracking [5], [6] allows an assessment of visual information extraction through time, and it has been used successfully in numerous studies investigating the extraction of visual and lexical information in text reading. This method has the advantage of leaving the stimulus unaltered and thus of not interfering with the normal reading process. However, its temporal resolution is insufficient to reveal the extraction of visual information in the recognition of individual words. In fact, the average duration of ocular fixation when reading English text (200–250 ms; [6]–[8]) is about the same as the time needed to recognize an isolated word (less than 250 ms; [9]). Accordingly, eye-tracking studies of reading have shown that short words are usually apprehended in a single eye fixation (i.e., mean saccade size is 7–9 letters; [6]).

Nevertheless, it is possible to manipulate the amount of information available during one fixation and to determine how this manipulation affects performance. Slowiaczek and Rayner [10] designed an experiment in which the letters surrounding the area fixated were masked simultaneously or sequentially. They showed no difference between the simultaneous and the sequential conditions when parafoveal processing was prevented, and suggested that their results indicate that letters are processed in parallel. Their sequential masking however, implicitly involved several assumptions about the properties of sequential processing: it was assumed that letter information extraction is performed at a rate of 10 ms per letter, and in a specific order. Any divergence between these assumptions and reality could prevent finding a difference between the results with simultaneous and sequential masking conditions. An alternative approach avoiding this problem would be to manipulate the availability of letter information systematically, such that the impact on performance of all the possible rates and orders of information extraction are evaluated.

Here, we propose to reveal the spatio-temporal course of information extraction during word identification by manipulating the availability of information in space and time in a systematic manner using a classification image technique. *Classification image techniques* have already been used to uncover the features involved in letter discrimination regardless of time [11]–[14]. They have also been used in the study of features involved at different moments in basic image discrimination [15], face identification [16], illusory shape discrimination [17], and letter identification [18]. They have, however, never been used to examine the reading of letter strings. Classification image methods are ideal for our purposes because their spatio-temporal resolution is only limited by the properties of the visual system and those of the equipment used to display the stimuli. Among the different classification image approaches available, we have chosen *Bubbles* [16], [19] because only *Bubbles* promises to render the *potent* visual information—the visual information that allows an observer to perform a particular task effectively. In contrast, reverse correlation, another classification image methods, promises to reveal the *represented* visual information, i.e. the visual information—effective and ineffective—that an observer relies upon to resolve a task [20]–[22].

**Figure 1:**
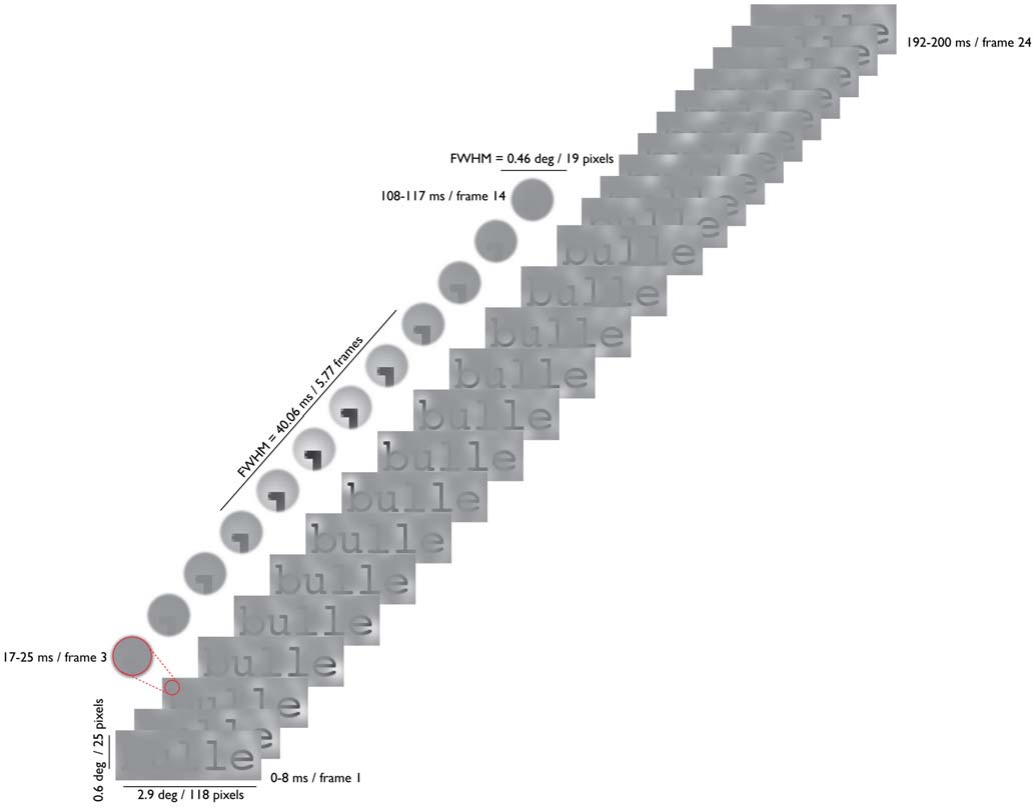
The French word “bulle” (“bubble” in English) sampled using 332 bubbles, which was the average number of bubbles used by the participants in the first half of the experimental sessions. Only the horizontal strip of the stimulus that contains letter signal is displayed. Each one of the 24 stimulus frames has a duration of 8.33 ms, for a total stimulus duration of 200 ms. The magnified portion of the stimulus shows a complete bubble cycle.

The following analogy illustrates how we applied the *Bubbles* technique to word reading. In the late stages of Emmenthal cheese production, a bacteria releases carbon dioxide gas and this process generates bubbles that become the famous holes. Imagine a word revealed by an animated sequence of masks very similar to a succession of thin, opaque slices of cheese cut from a brick of Emmenthal. This is in essence what we did in the present experiment: On each trial, a target word was randomly sampled across space and time by a collection of tridimensional (i.e., height, width, and time) Gaussian windows (or bubbles; see Figure 1 and Movies S1 and S2).

Thus, on any given trial, several masks (or slices of cheese, to pursue our analogy) were successively placed over a target letter string to modulate the availability of visual information (partial or complete letters) across time. One fundamental feature of *Bubbles* is that the sampling of the stimulus on any given trial is entirely random. This involves a significant cost in terms of the number of trials required to uncover information use (e.g., we ran a total of 50,000 trials in the present experiment). This cost however, is offset by a major advantage: the systematic and unbiased search of space-time use of information during reading.

## Methods

### Participants

Ten students from the Université de Montréal with normal or corrected-to-normal visual acuity took part in the experiment. All procedures were carried out with the ethics approval of the Université de Montréal.

### Materials and stimuli

Stimuli were displayed on a high-resolution Sony monitor with a refresh rate of 120 Hz. The experiment ran on a Macintosh G4 computer. The experimental program was written in Matlab, using functions from the Psychophysics Toolbox [23], [24]. The viewing distance was maintained constant at 91 cm by using a chinrest. Stimuli were lowercase words printed in Courier 40 point. They appeared in dark grey over a light grey background (fixed luminance of 64.8 cd/m^2^; minimum and maximum luminances were 0.48 and 130 cd/m^2^, respectively). The luminance contrast of the stimulus was adjusted according to the criteria and procedure described below.

Stimuli were constructed from a list of 1,000 five-letter French words. We chose five-letter words because they are relatively taxing for the visual system while clearly remaining within the alleged parallel-processing boundary (i.e., a small but systematic word-length effect is observed for words made of seven letters or more; [2]–[4]). These five-letter words subtended a vertical x horizontal spatial extent of 9.8×46.1 mm (0.6×2.9 deg of visual angle or 25×118 pixels). The list of words was constructed using BRULEX, a lexical database for French [25], and it excluded all words with diacritic marks (e.g., é, ê, à, and so on). We also discarded extremely low frequency words with which the participants may have been unfamiliar.

The stimuli consisted of movies made from a sequence of 24 frames shown at a rate of 120 Hz (total stimulus duration of 200 ms; this duration is less than the time required to plan and execute an eye saccade). On any given trial, a randomly selected word was sampled using Gaussian apertures (bubbles) randomly positioned in space-time. Each bubble had a full-width half maximum of 0.46 degree of visual angle (or 19 pixels) in the spatial domain and a full-width half maximum of 48 ms (5.77 frames), in the temporal domain (see Figure 1 and Movies S1 and S2). This is less than the estimated time required to execute an unvoluntary attentional saccade, which ranges between 50 and 84 ms [26], [27]. It was thus difficult for the readers to adjust on-line to the position of the bubbles because they appear briefly and at random locations. The number of bubbles was adjusted twice during the experiment, as will be explained shortly.

### Procedure

Each participant completed a total of 5,000 trials divided in 20 experimental sessions (250 trials each) spread over 10 weeks on average. Reading accuracy was maintained at 51% correct. In a Bubbles experiment, the usual procedure is to maintain accuracy at a fixed level by adjusting the number of bubbles on a trial-by-trial basis to make the task more difficult (or easier) when the accuracy of the subject is higher (or lower) than the targeted accuracy (e.g., [19]). A trial-by-trial modification of the number of bubbles, however, requires the online computation of each dynamic stimulus. In the present experiment, this computation took on average 8 seconds, which was impractical. Therefore, we opted for a dual strategy to maintain performance at a fixed level while avoiding excessively long inter-trial intervals.

We estimated the number of bubbles required by each participant to read accurately on 51% of trials in two 50-trial *setting sessions*, one before the first *experimental session* and the other before the eleventh *experimental session*. In these setting sessions, the luminance contrast of the stimuli was kept constant at 0.1, and the number of bubbles was adjusted on a trial-by-trial basis using a gradient descent procedure [28]. In the experimental sessions, performance was maintained at 51% correct by adjusting the luminance contrast of the stimulus on a trial-by-trial basis using QUEST [29], with the initial contrast being set at 0.1 for all participants. This procedure allowed us to generate the bubbles' masks for each experimental trial prior to the experimental sessions, thus reducing the time required to prepare the upcoming trial to approximately 4 s, which was relatively comfortable for participants.

The sequence of events for each trial in the *experimental sessions* was identical to that of the *setting sessions*. On each trial a homogenous grey screen was first displayed for 250 ms and a 1300 Hz pure tone accompanied this grey screen for the first 122 ms. The grey screen was immediately followed by a dynamically bubbled word displayed for 200 ms at the center of the screen. This was in turn immediately followed by a homogenous grey screen that remained visible until the participant responded. The participant's task was to read the target word aloud as quickly and as accurately as possible. A response key triggered a dialog box into which the subject typed his/her response using the appropriate computer keyboard keys followed by the «ENTER» key. No feedback was provided to participants.

## Results

In the first half of the experiment, a mean of 332.4 bubbles (between 222 and 530 across participants) with an average Weber contrast of 0.094 (the average contrast was of 0.1 and 0.088 at the beginning and the end, respectively) was necessary to maintain performance at 51% correct. (The French word “bulle” (“bubble” in English) displayed in Figure 1 is revealed by 332 bubbles.) In the second half of the experiment, the corresponding numbers were 252.3 bubbles (between 172 and 455 across participants) with an average Weber contrast of 0.099 (the average contrast was of 0.1 and 0.098 at the beginning and the end, respectively).

The efficient use of the spatio-temporal information in the stimulus was determined by performing a multiple linear regression on the bubbles masks (explanatory variables) and the participant's response accuracy (predictor variable). First, we constructed one regression coefficient volume per session and per subject by subtracting the sum of the bubbles' masks that led to an incorrect response from the sum of the bubbles' masks that led to a correct response. These volumes of regression coefficients will be referred to as *classification movies*, which is a natural extension of *classification image*, a term widely used to refer to planes of regression coefficients (e.g., [30]). The elements of these movies will be referred to as *voxels*. Second, we constructed a group classification movie that combined the classification movies of all subjects, weighted by the number of bubbles used. If all parts of the stimulus (the various letters) were of equal importance for success in the task (word identification), the voxels would be uniform. Any local divergence from uniformity indicates that this particular part of the stimulus (in space-time) was particularly important for the task at hand. The statistical analysis was restricted to the central horizontal strip one third the height of the group classification movie (43×128 pixels), which approximately represents the area occupied by the word. The remainder of the group classification movie was used to estimate the mean and the standard deviation of the null hypothesis, and to Z-score the group classification movie. Finally, we conducted a one-tailed Pixel test [31] on the group classification movie (*S_r_* = 132,096 voxels; full-width half maximum = 12.696; *Z_crit_* = 4.168; *p*<.025).

**Figure 2:**
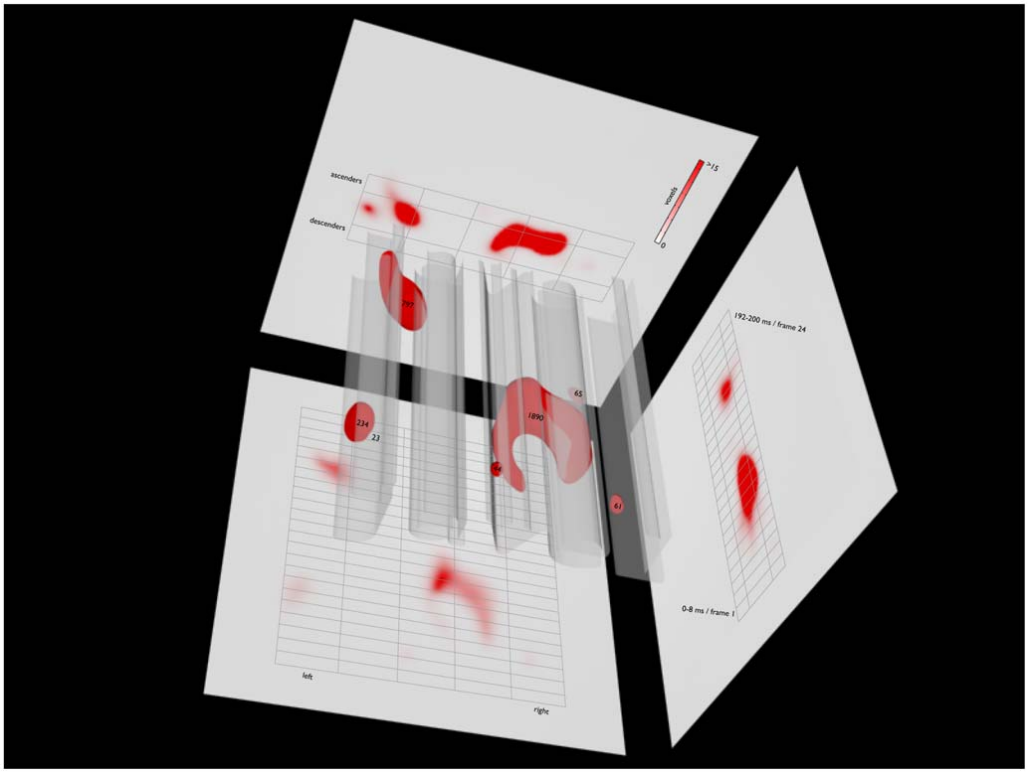
Thresholded classification movie. The space-time voxels reaching statistical significance are depicted in bright red in the center of the figure and are overlaid on the word “javel”. The numbers within or nearest to each of the seven blobs indicate the size of these blobs in voxels. The voxels were projected onto three bidimensional planes: the back wall—to isolate the spatial left-right and up-down dimensions; the floor—to isolate the time and the left-right dimensions; and the right wall—to isolate the time and the up-down dimensions. The number of significant voxels projected onto a single pixel on the planes is represented by red saturation (see legend). The dim grey lines delimit the 24 frames on the time dimension and the three different regions of the five letters on the space dimensions (i.e., body, ascenders, descenders).

Figure 2 shows the thresholded classification movie in a rich tridimensional graphic (see also Movies S3 and S4). The space-time voxels reaching statistical significance are depicted in bright red in the center of the figure and are overlaid on the word “javel”. Four relatively small blobs (<66 voxels) and, more importantly, three larger blobs can clearly be seen. The largest blob (1890 voxels) is shaped like a croissant, the second largest (797 voxels) resembles a bottle, and the third largest is almost round, looking more or less like a sugared almond (234 voxels). Note that the raw significant voxels were convolved with a Gaussian kernel with a full width half maximum of 2.35 pixels.

This tridimensional representation ultimately comes short of fully conveying the exact shape and location of the blobs in space-time. To remedy this problem, the significant voxels were projected onto three bidimensional planes: the back wall—to isolate the spatial left-right and up-down dimensions; the floor—to isolate the time and the left-right dimensions; and the right wall—to isolate the time and the up-down dimensions. The number of significant voxels projected onto a single pixel on the planes is represented by red saturation (see legend). To further facilitate space-time localization, we have added light grey lines delimiting the 24 frames on the time dimension and the three different regions of the letters on the space dimensions (i.e., body, ascenders, descenders).

A glance at the right wall reveals two moments especially correlated with accurate reading: one between frames 5 and 10 and the other between frames 19 and 21. It also shows that most of the voxels correlated with reading accuracy are located in the top half of the body region and, to a lesser extent, in the ascender region. Interestingly, there are no significant voxels in the descender regions. Looking at the back wall, it can be seen that the letter positions most correlated with reading accuracy are 4 (letter positions are numbered from 1—leftmost—to 5—rightmost), followed by 1, then 3, then 2, and, finally, 5. The floor helps to visualize the interactions between the importance of letter positions and time in reading accuracy.

To summarize, the space-time use of letter positions 3 and 4 forms the large croissant-shaped blob. It begins around frame 4 (25–33 ms) or 5 (33–42 ms) and ends around frame 14 (108–117 ms) or 15 (117–125 ms). The early space-time use of letter position 1 forms the sugared almond-shaped blob. It approximately extends from frame 5 (33–42 ms) to frame 8 (58–67 ms). Together, the croissant- and the sugared almond-shaped blobs are responsible for the first burst of activity that can be distinctively seen on the right wall. The second burst of activity on the right wall is caused by the bottle-shaped blob. It corresponds to the effective use of letter positions 1 and 2 between frames 17 (133–142 ms) and 20 (158–167 ms).

### Ideal Reader Analysis

We elaborated an ideal reader to reveal the overall optimal strategy of information use. For each word of the lexicon, the ideal reader selected, on successive processing cycles (*c*), the reading strategy (*S_c_*) that reduced the most the uncertainty regarding the identity of the target word based on its perfect lexical knowledge and given the letter information already accumulated on the preceding processing cycle(s). The optimal reading strategy *S_c_* at clock cycle *c* was calculated as follows:
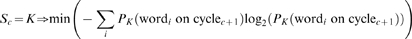
where *P_K_*(word*_i_* on cycle *_c_*_+1_) is the probability that each word in the lexicon is the target given a particular reading strategy *K* on cycle *c*+1. Since all five-letter words had equal probability of being selected during the experiment, the probability that a given word was the target did not need to be scaled by its lexical frequency. What warrants the optimality of the ideal reader is that it exhaustively searches the possible reading strategies for the processing of cycle *c*+1; that is, all the sets of *m* letters (consecutive or not) in words of *n* letters are considered, and the one that minimises entropy one cycle ahead is selected for processing. This resulted in one optimal strategy for each word that was given as input to the ideal reader. The overall optimal reading strategy is the average of all the optimal reading strategies.

We ran the ideal reader on the list of five-letter words used in our experiment as well as on the lists of all four-, five-, six- and seven-letter French and English words. Changing the processing capacity limit (*m*) of the ideal reader from one to four letters per cycle made little difference in the overall optimal strategy (at least once noise was introduced in the process); thus we only report the results obtained with the ideal reader with a processing capacity of one letter per cycle (without noise). Tables 1 and 2 give the mean of the overall optimal reading strategies across processing cycles made to sum to one. Interestingly, approximately three letters are required on average to identify words irrespective of word length and language. We will return to this observation as well as to the main findings of the ideal observer analysis in the Discussion.

**Table 1:** Relative importance of each letter position in French words containing 4, 5, 6 or 7 letters.

Word Length	Letter 1	Letter 2	Letter 3	Letter 4	Letter 5	Letter 6	Letter 7	Average number of letters	Center of gravity
4	0.297	0.199	0.243	0.262				2.676	2.470
5 (a)	0.265	0.169	0.220	0.201	0.149			2.750	2.794
5 (b)	0.257	0.169	0.222	0.202	0.151			2.936	2.821
6	0.220	0.141	0.187	0.180	0.149	0.123		2.991	3.266
7	0.189	0.126	0.169	0.156	0.138	0.128	0.093	2.995	3.686

For five-letter words, list (a) contained all five-letter words without diacritics whereas list (b) contained the 1000 words used in the bubbles experiment reported in the present article. The center of gravity of the relative importance of letter position and average number of letter required to identify words are also provided.

**Table 2:** Relative importance of each letter position in English words containing 4, 5, 6 or 7 letters.

Word Length	Letter 1	Letter 2	Letter 3	Letter 4	Letter 5	Letter 6	Letter 7	Average number of letters	Center of gravity
4	0.289	0.210	0.244	0.257				3.043	2.469
5	0.233	0.166	0.211	0.199	0.191			2.966	2.950
6	0.212	0.142	0.174	0.178	0.138	0.156		2.939	3.359
7	0.184	0.129	0.159	0.154	0.129	0.119	0.126	2.829	3.777

The center of gravity of the relative importance of letter position and average number of letter required to identify words are also provided.

## Discussion

We used the *Bubbles* technique to examine the extraction of visual information over the first 200 ms in a five-letter word reading task. To our knowledge, our study is the first to reveal the spatio-temporal course of information extraction during word identification at such a fine time scale. In a nutshell, we found that letter positions 3 and 4 are favored between about 25 ms and 125 ms after stimulus onset. Letter position 1 is favored between about 33 ms and 67 ms after stimulus onset. Letter position 1 is favored a second time, together with letter position 2, between about 133 ms and 167 ms.

Our results are congruent with a number of findings reported in the literature. First, an analysis restricted to the temporal dimension of our results revealed some oscillations in use of letter information across time. We calculated, on each frame, the mean z-score on a central horizontal strip spanning one third the height of the group classification movie (43×128 pixels; this corresponds approximately to the area occupied by the word). A Fourier transform was then performed on the resulting temporal vector of information use, and revealed a peak of energy at 10 Hz. This is in agreement with recent discussions that have related the appearance of discrete “perceptual” [16], [32], [33] and “cognitive” [34] moments to oscillations broadly identified as covering a range between 6 and 15 Hz.

Second, an exact binomial sign test indicated that the upper portion of the letters contains more significant voxels than the lower portion (*N_upper_* = 2211; *N_lower_* = 903; *p*<001). In fact, 94% of the significant pixels falling on letter extensions were located on the ascenders, further supporting the bias for the upper part of words. This is consistent with the findings of Huey [35], which showed that reading is slower and more effortful when the top half of words is removed than when the bottom half of words is removed. Intriguingly, this bias for the upper portion of the letters appears to be restricted to the case where letters are presented within the context of words. Fiset et al. [14] have examined the diagnostic features for the identification of isolated letters using a similar classification-image technique as the one presented in this paper, and found that forty-one percent of the significant pixels were located in the upper half of lowercase letters. The divergence between our results and those of Fiset et al. [14], [18] suggests that letter representations may be slightly different for isolated letters and for letters in words.

One novel aspect of our results is the particular usefulness of letter positions one, three, and four for five-letter word identification. The proportions of significant pixels falling on letter positions one to five are, respectively, 0.271, 0.067, 0.255, 0.381, and 0.026. An ideal reader analysis revealed that letter position one, three, and four are indeed the most informative in five-letter words. The utilization of these most informative letter positions by the human readers may reflect a strategy of information extraction whereby more resources are allocated to the most informative letters. However, letter position 1, which is the most informative, is employed less effectively by human readers than letter position 4. This, in turn, suggests that letter position effectiveness for human readers is due partly to constraints imposed by properties of the human visual system rather than to constraints that are exclusively determined by the lexicon.

We also ran the ideal reader with four- to seven-letter French and English words. Surprisingly, the number of letters needed by the ideal reader to identify a word was constant across lengths—three letters were required on average. This suggests a new hypothesis for the lack of word length effect in skilled readers for words containing four to seven letters. The fact that the number of letters in a word hardly has any effect on the time required to read it [4] has often been taken to support the idea that normal word recognition rests upon spatially parallel letter processing (i.e. all letters are processed simultaneously). Our classification movie is incompatible with such a fully parallel reading strategy. Indeed, there is no time interval during which all the letters of the stimulus are processed simultaneously and there is a clear modulation of the relative importance of the five letter positions across time; this argument holds for raw Z-scores as well. Both of these facts are incompatible with a fully parallel model. However, the lack of a word length effect would also be predicted by a serial process if the number of letters that needs to be processed for word recognition remains constant across word lengths. This hypothesis may also, to a large extent, explain the occurrence of a substantial length effect in pseudoword reading [4]. Indeed, the number of legal pseudowords of a given length is necessarily greater than the total number of words of that length and this number grows faster with increasing length with pseudowords than with words. Because of this, more letters would need to be identified to read pseudowords than words and this difference should be magnified with letter strings of increased length.

Our classification movie is also compatible with a partially parallel reading strategy, where the most informative letters are sometimes processed simultaneously. For instance, in the classification movie, the letter positions 3 and 4 appear to be simultaneously useful for a while. Unfortunately, we cannot distinguish between the partially parallel and the serial strategy based on this analysis. Even though two or more letters may appear to have been processed simultaneously in the thresholded classification movie (Figure 2), it should be kept in mind that the group classification movie was elaborated from the weighted sum of all bubble masks. This implies that two letters that are revealed simultaneously in the results may have actually been used independently on different trials. To verify if letter positions were used independently or simultaneously, we conducted a conjunction analysis (i.e. an analysis where we verify how the simultaneous availability of each possible combinations of letter position affected performance on each frame). A partially parallel strategy would predict that when some combinations of letter positions (e.g. letter positions 3 and 4) are simultaneously revealed, the performance should be higher than when only one letter from that combination (e.g. 3) is revealed. No combination of letter positions led to such a significant performance improvement. However, the absence of a significant effect may be due to a lack of statistical power; we performed a total of 240 independent z-tests (i.e. all within-frame pairwise letter-position combinations) and, therefore, we had to apply a stringent Bonferroni correction on the *p*-values. Clearly more empirical work will be needed to fully uncover how we read between eye saccades and to test the new hypothesis that it may be sequential and nearly optimal. We believe that the Bubbles technique will play an important role in this endeavour.

## Supporting Information

Movie S1 The French word “bulle” (“bubble” in English) sampled using 332 bubbles, which was the average number of bubbles used by the participants in the first half of the experimental sessions. Each one of the 24 frames has a duration of 333.33 ms, for a total duration of 8 s (40 times slower than in the experiment).
(0.08 MB MOV)

Movie S2 The French word “bulle” (“bubble” in English) sampled using 332 bubbles, which was the average number of bubbles used by the participants in the first half of the experimental sessions. Each one of the 24 frames has a duration of 8.33 ms, for a total duration of 200 ms (same as in the experiment).
(0.08 MB MOV)

Movie S3 Thresholded classification movie. The space-time voxels reaching statistical significance are depicted in bright red in the center of the figure and are overlaid on the word “javel”. Each one of the 24 frames has a duration of 333.33 ms, for a total duration of 8 s (40 times slower than in the experiment).
(0.10 MB MOV)

Movie S4 Thresholded classification movie. The space-time voxels reaching statistical significance are depicted in bright red in the center of the figure and are overlaid on the word “javel”. Each one of the 24 frames has a duration of 8.33 ms, for a total duration of 200 ms (same as in the experiment).
(0.10 MB MOV)
